# Sustained Inflammatory Signalling through Stat1/Stat2/IRF9 Is Associated with Amoeboid Phenotype of Melanoma Cells

**DOI:** 10.3390/cancers12092450

**Published:** 2020-08-28

**Authors:** Aneta Gandalovičová, Anna-Marie Šůchová, Vladimír Čermák, Ladislav Merta, Daniel Rösel, Jan Brábek

**Affiliations:** 1 Department of Cell Biology, Charles University, 12843 Prague, Czech Republic; aneta.gandalovicova@natur.cuni.cz (A.G.); amsuchova@gmail.com (A.-M.Š.); vladimir.cermak@natur.cuni.cz (V.Č.); ladislav.merta@natur.cuni.cz (L.M.); rosel@natur.cuni.cz (D.R.); 2 Biotechnology and Biomedicine Centre of the Academy of Sciences and Charles University (BIOCEV), 25242 Vestec, Czech Republic

**Keywords:** plasticity, melanoma, amoeboid, inflammation, interferon, invasion, mesenchymal

## Abstract

**Simple Summary:**

Treatment of metastatic cancer is complicated by the ability of cancer cells to utilize various invasion modes when spreading through the body. Here, we studied the transition of melanoma cells between the round, amoeboid and elongated, mesenchymal invasion modes. Our results show that inflammatory signalling, which is commonly upregulated in the tumour microenvironment, is associated with the amoeboid phenotype of cancer cells. Treatment of melanoma cells with interferon beta promotes the amoeboid invasion modes and individual invasion. This suggests that inflammation associated signalling contributes to cancer cell invasion plasticity.

**Abstract:**

The invasive behaviour of cancer cells underlies metastatic dissemination; however, due to the large plasticity of invasion modes, it is challenging to target. It is now widely accepted that various secreted cytokines modulate the tumour microenvironment and pro-inflammatory signalling can promote tumour progression. Here, we report that cells after mesenchymal–amoeboid transition show the increased expression of genes associated with the type I interferon response. Moreover, the sustained activation of type I interferon signalling in response to IFNβ mediated by the Stat1/Stat2/IRF9 complex enhances the round amoeboid phenotype in melanoma cells, whereas its downregulation by various approaches promotes the mesenchymal invasive phenotype. Overall, we demonstrate that interferon signalling is associated with the amoeboid phenotype of cancer cells and suggest a novel role of IFNβ in promoting cancer invasion plasticity, aside from its known role as a tumour suppressor.

## 1. Introduction

Cancer mortality is mostly due to the invasive potential of tumour cells and their ability to establish secondary tumours—i.e., metastases. However, the metastatic behaviour of cells and associated invasion plasticity are largely heterogenous, making them difficult to target. Thus, further understanding of these processes is important for the development of migrastatic therapies [1,2].

The cancer cells’ invasive behaviour is largely dictated by the tumour microenvironment (TME), comprising various cell types that mutually interact. Typically, this promotes the production of various secreted molecules that can alter the behaviour of cells in the tumour stroma and create an inflammatory-like environment, which is considered a tumour hallmark [3,4]. Moreover, the physical properties of the extracellular matrix (ECM), such as pore size or density, determine the invasive ability of cells [5,6].

In response to these cues, cancer cells adapt their invasive behaviour by switching among invasion modes [7,8]. Cells can invade collectively in the form of whole sheets, strands or cell clusters by undergoing partial epithelial–mesenchymal transition while maintaining cell–cell junctions [9]. Additionally, cells can also disrupt intercellular adhesions and invade individually, using either the mesenchymal or amoeboid mode. Mesenchymally invading cells make use of proteolytic degradation of the ECM and integrin-based cell–ECM adhesions, which results in their typical elongated shape with cell protrusions at the leading edge [10,11]. In turn, amoeboid cells are mostly seen as round cells with membrane blebs, that dynamically deform the cell body to squeeze through pores within the ECM [12,13]. Importantly, cells can switch among these modes in response to conditions of the TME, such as matrix density or reciprocal signalling within the stroma, referred to as mesenchymal–amoeboid transition (MAT) and amoeboid–mesenchymal transition (AMT) [14,15].

To understand the plasticity of cancer invasion, approaches to induce MAT and AMT have been investigated [8,14,16,17]. Modifying the activity of Rho GTPases, which are key regulators of the invasion modes, can drive both AMT and MAT. RhoA activation promotes the amoeboid phenotype, while the use of ROCK inhibitors induces cell elongation and mesenchymal invasion. Accordingly, the upregulation of IL6/Stat3 or IL-1α/NF-κB signalling, leading to RhoA activation, has been implicated in promoting and maintaining the amoeboid phenotype in melanoma cells [18,19].

Pro-inflammatory signalling has been associated with phenotypic plasticity in melanoma [20]. One of the key signalling pathways associated with inflammation is the JAK/STAT signalling cascade, activated in response to interferons type I (IFNs α, β, ω) or type II (IFNγ). Upon type I IFN binding to its receptor, Janus kinase 1 (Jak1) and tyrosine kinase 2 (Tyk2) phosphorylate signal transducer and activator of transduction 1 and 2 (Stat1 and Stat2), which dimerize and bind IRF9, resulting in the formation of the Stat1/Stat2/IRF9 complex (referred to as ISGF3) [21]. The type II IFN response is mediated by Stat1 homodimers [22]. Secretion of IFNs is generally activated in response to viral infection but is also induced in response to various stress factors, and cancers often show an intrinsic IFN signature [23,24]. This modulates cancer immunosurveillance and response to therapy. IFN-β is widely used as an adjuvant for melanoma therapy and has been shown to prolong overall and relapse-free survival [25]. Moreover, IFN-β increases tumour infiltration by immune cell subsets, including CD4+ and CD8+ T-cells [26]. Consistently, IFN-β treatment was shown to prolong survival and increase T-cell recruitment into the tumour tissue of B16F10-bearing mice, which could contribute to the therapeutic efficacy of immune checkpoint inhibitors [27]. Peritumourally administered IFN-β enhances the antitumor effect of anti-PD-1 antibody against B16F10 melanoma [28].

In this study, we demonstrate that pro-inflammatory signalling mediated by IFNβ is associated with the amoeboid phenotype of cancer cells and suggest a novel role of IFNβ in promoting cancer invasion plasticity, aside from its known role as a tumour suppressor.

## 2. Results

### 2.1. Data Analysis Reveals Upregulation of Inflammation-Associated Genes after MAT

**Figure 1:**
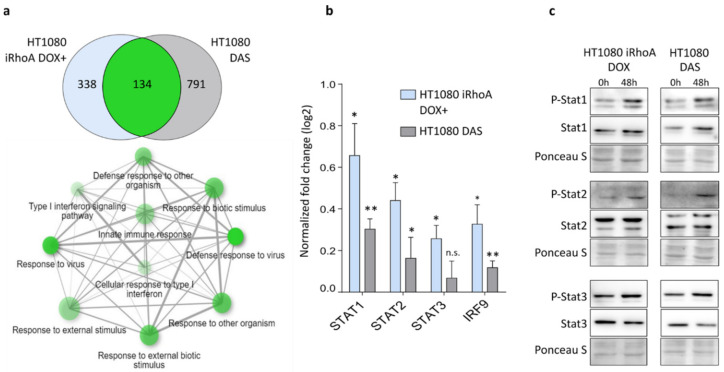
Interferon response-like signalling is upregulated in amoeboid cells. (**a**) Ten most significant GO Biological Process terms enriched in the genes affected concordantly by both iRhoA and dasatinib (DAS) treatment. Only transcripts with adjusted *p*-value ≤ 0.25 and fold change >1.5 in either direction were considered differentially expressed. Network plot was generated using ShinyGO v0.61 online tool [30]. (**b**) Gene expression (log2 fold change) of STAT1, STAT2, STAT3 and IRF9 in cells after mesenchymal–amoeboid transition (MAT) in 3D collagen (48 h) determined by RT-qPCR, normalized to control cells without MAT induction. (**c**) Immunoblotting detection of Stat transcription factors Stat1, Stat2 and Stat3 in HT1080 cells before and after MAT. *p*-values: ** *p* < 0.01, * *p* < 0.05. Detailed information about Western blot can be found in Figure S1.

To determine amoeboid phenotype associated signalling, we analysed previously published transcriptomic data from human fibrosarcoma HT1080 cells in 3D collagen before and after MAT induced either by the expression of doxycycline-inducible constitutively active RhoA (iRhoA) or by the treatment of the cells with a Src/Abl kinase inhibitor dasatinib (DAS) [16]. To identify functional gene categories upregulated in amoeboid cells, we performed an enrichment analysis for Gene Ontology (GO) Biological Process terms on genes affected by both treatments [29]. We revealed a strong similarity in the spectra of upregulated genes to type I IFN response (Figure 1a). To affirm that type I IFN signalling is elevated in amoeboid cells, we measured the expression of genes encoding Stat transcription factors (TFs) and IRF9, a component of ISGF3, by RT-qPCR in HT1080 cells undergoing MAT induced by both iRhoA expression and DAS treatment in a 3D environment (Figure 1b). We detected significantly increased levels of STAT1, STAT2 and IRF9 transcripts after both treatments inducing MAT. Next, we determined the activation status of the corresponding Stat TF proteins measured as phosphorylation detected by immunoblotting, which revealed a predominant activation of Stat1/2 compared to Stat3, in agreement with the upregulation of type I IFN response identified in the transcriptomic data (Figure 1c).

### 2.2. Inflammation-Associated Signalling Affects Invasion Plasticity in Melanoma Models

**Figure 2:**
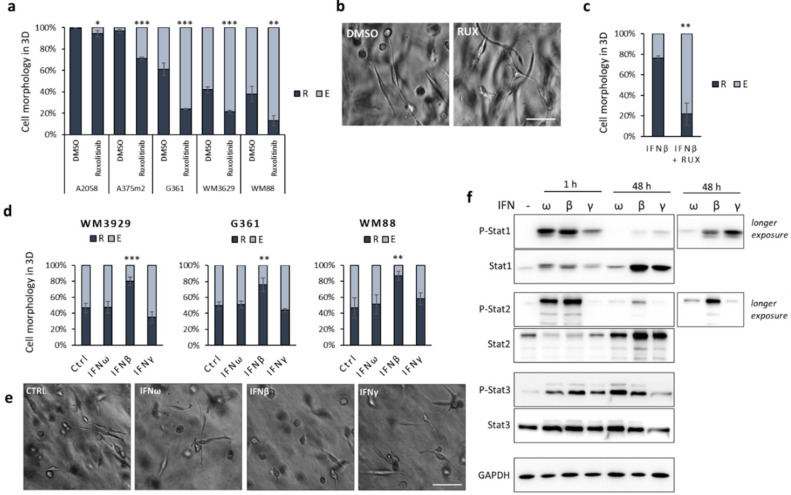
Role of IFN signalling in melanoma invasion plasticity. (**a**) Inhibition of Jak1/2 by Ruxolitinib promotes the elongated, mesenchymal phenotype of melanoma cells cultured in 3D collagen (48 h). (**b**) Representative image of WM3629 cells after 48 h in 3D collagen treated with DMSO or Ruxolitinib. (**c**) Quantification of morphology of WM3629 cells treated with IFNβ alone or IFNβ plus Ruxolitinib after 48 h in collagen. (**d**) Quantification of morphology of melanoma cells cultured in 3D collagen for 48 h after treatment with IFNs (overall exposure to IFNs took 96 h). (**e**) Representative images of WM3629 cells after 48 h in 3D collagen treated with IFNs. (**f**) Immunoblotting detection of Stat transcription factors Stat1, Stat2 and Stat3 activation after 1 h and 48 h IFN treatment in WM3629 cells. Scale bar 100 µm in both (**b**) and (**e**). R = round, E = elongated. *p*-values: *** *p* < 0.001, ** *p* < 0.01, * *p* < 0.05. Detailed information about Western blot can be found at Figure S4.

Transcriptomic analysis and the subsequent data validation of genes upregulated after MAT suggested that amoeboid cells display intrinsically upregulated type I IFN signalling. To study the role of IFN signalling in invasion plasticity further, we focused on human melanoma cell lines, since they are known to exhibit high inherent invasion plasticity governed by autocrine and paracrine production of various cytokines [31,32,33]. Initially, we tested the effect of IFN signalling suppression by Ruxolitinib, a Jak1/2 inhibitor, on a panel of amoeboid and mixed-morphology melanoma cells lines. The inhibition of Jak1/2 significantly promoted the elongated, mesenchymal migratory phenotype of five tested melanoma cell lines in 3D collagen (Figure 2a,b). Next, we tested the effect of IFN signalling activation on three selected cell lines with mixed morphology—namely WM3629, G361 and WM88. We treated the cells with IFNs of both type I (IFNω and β) and type II (IFNγ). Interestingly, IFNβ—but neither IFNω nor IFNγ—promoted the round amoeboid phenotype in all three cell lines (Figure 2d,e), and this could be blocked by Ruxolitinib (Figure 2c). To compare the activity of all three IFNs and disclose their differing effect on cell morphology, we assessed the phosphorylation levels of Stat1, 2 and 3 at different time points (Figure 2f; Figures S2a and S3). Only IFNβ induced a long-term response, observed as the prolonged phosphorylation of Stat1 and Stat2, but interestingly also as the accumulation of Stat1 and Stat2 proteins, which are known to sustain inflammatory signalling [34]. To exclude that the round phenotype observed in response to IFNβ is caused by the induction of apoptosis, we measured cell proliferation in the 3D collagen of untreated and treated cells and detected a decrease consistent with the anti-proliferative effects of IFNβ (Figure S2c), but no significant differences in numbers of dead cells were detected (Figure S2d). Moreover, by live cell imaging of cells in 3D collagen, we documented that IFNβ treated cells invade almost exclusively as round, amoeboid cells (Video S1).

### 2.3. IFNβ Treated Cells Upregulate Expression of Pro-Invasive Cytokines and Increase Individual Invasion

**Figure 3:**
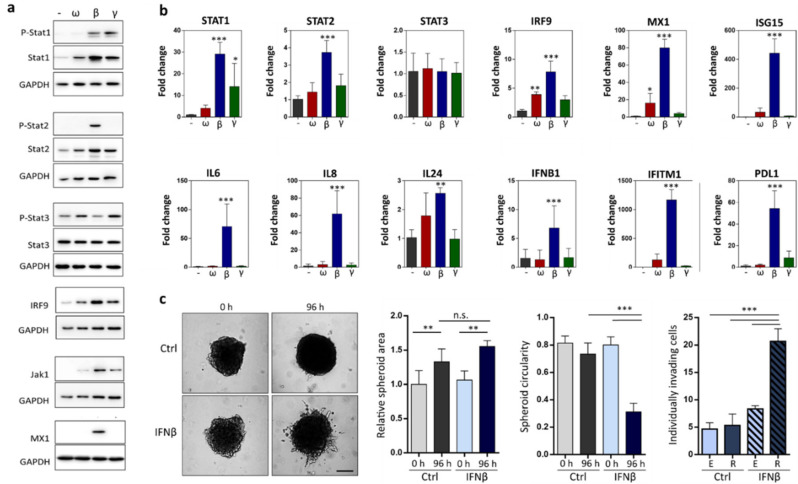
Characterization of IFN treated cells. (**a**) Immunoblotting detection of proteins from WM3629 cells cultured in 3D collagen for 48 h after treatment with IFNs (overall exposure to IFNs was 96 h). (**b**) Gene expression changes in WM3629 cells cultured in 3D collagen for 48 h (overall exposure to IFNs 96 h). (**c**) Spheroids of WM3629 cells, quantification of spheroid area, circularity, and the number of individually invading cells after 96 hours in 3D collagen. Scale bar 500 µm. R = round, E = elongated. *p*-values: *** *p* < 0.001, ** *p* < 0.01, * *p* < 0.05. Detailed information about Western blot can be found at Figure S5.

To gain insight into the role of IFN signalling in cancer cell invasion plasticity, we prepared protein and RNA lysates from WM3629 cells after IFN treatment in 3D collagen and analysed the expression of Stat TFs and downstream regulated proteins/genes (Figure 3a,b). Both Stat1 and Stat2 showed statistically significant increases in gene expression levels, and at protein level we confirmed the upregulation of both phosphorylated and total proteins after treatment with IFNβ. We further tested the expression levels of IRF9, the adaptor protein important for binding Stat1/2 heterodimers in the ISGF3 complex, and indeed confirmed its upregulation at both protein and transcript levels in IFNβ treated cells. Interestingly, the IRF9 levels show similar upregulation time course as Stat1, Stat2, as well as Jak1 (Figure S2b and Figure S3). This points to the role of the long-term type I IFN response in promoting the amoeboid phenotype. IFNβ also increased the expression of MX1, ISG15, IFITM1 and PDL1, known interferon responsive genes, and of genes encoding secreted cytokines IL6, IL8 and IL24. We also detected the increased expression of IFNB1 gene in response to IFNβ, suggesting a positive feedback loop, maintaining the sustained IFN signalling. Since we detected the upregulation of pro-invasive molecules (MX1, IFITM1, IL6, IL8), we sought to investigate the effect of IFNβ on cell invasion from multicellular spheroids. We cultivated cell spheroids in control media and in the presence of IFNβ, and then tested their invasion in 3D collagen. Interestingly, IFNβ pre-treatment did not increase the overall size of the spheroids, but increased the number of individually migrating cells, most of which acquired the amoeboid invasion phenotype (Figure 3c).

### 2.4. Suppression of IFN Signalling by SOCS1 Expression or IRF9 Knockdown Promotes the Mesenchymal Phenotype

**Figure 4:**
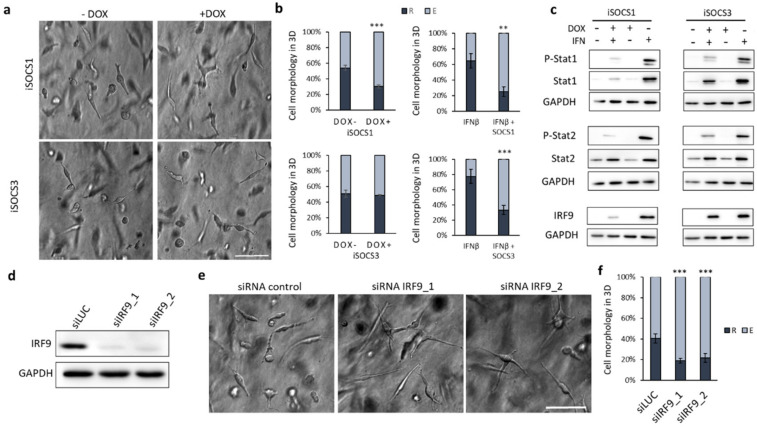
Downregulation of IFN signalling by IRF9 knockdown or SOCS1 expression promotes the mesenchymal phenotype. (**a**) Representative image of WM3629 after 48 h in 3D collagen expressing inducible SOCS1 and SOCS3 (iSOCS1 and iSOCS3). (**b**) Quantification of morphology of WM3629 cells with induced expression of iSOCS1 and iSOCS3 alone (left) and pre-treated with IFNβ (right) cultured in 3D collagen for 48 h (overall exposure to IFNβ 96 h). (**c**) Immunoblotting for proteins in samples of WM3629 cells with iSOCS1 and iSOCS3 expression treated with doxycycline (DOX) and/or IFNβ (48 h). (**d**) Downregulation of IRF9 after siRNA KD verified by immunoblotting. (**e**) Representative image of WM3629 cells in 3D collagen treated with siRNA. (**f**) Quantification of morphology of WM3629 cells with siRNA KD of IRF9 in 3D collagen. Scale bar 100 µm. R = round, E = elongated. *p*-values: *** *p* < 0.001, ** *p* < 0.01. Detailed information about Western blot can be found at Figure S8.

In cells, the fine tuning of the JAK/STAT signalling is naturally provided by the suppressor of cytokine signalling (SOCS) proteins, which function as negative regulators of the signalling pathway. To determine whether the overexpression of the SOCS proteins can directly affect melanoma invasion and/or suppress the effect of IFNβ, we prepared stable cell lines bearing EGFP-tagged doxycycline-inducible SOCS1 and SOCS3 (iSOCS1 and iSOCS3) gene expression constructs (Figure S6a). We confirmed the high efficacy of iSOCS1 and iSOCS3 proteins by testing their ability to block interferon-induced phosphorylation of Stat1 and Stat2 proteins (Figures S3 and S6b). The induced expression of both SOCS1 and SOCS3 was able to block the IFNβ mediated round phenotype (Figure 4b); however, only SOCS1 altered WM3629 morphology directly by promoting the mesenchymal phenotype (Figure 4a,b). To study this further, we analysed cell lysates from WM3629 control cells and cells with induced expression of iSOCS1 and iSOCS3 untreated and treated with IFNβ. We revealed that, unlike SOCS3, SOCS1 decreases the total level of Stat1, but also IRF9, providing an explanation for its stronger pro-mesenchymal activity (Figure 4c). Accordingly, the induced expression of SOCS1, but not SOCS3, was able to prevent HT1080 cells from undergoing MAT in response to DAS (Figures S3 and S7).

After demonstrating that SOCS1 decreases IRF9 protein levels, we went further and investigated the effect of siRNA knockdown (KD) of IRF9. Using two different siRNAs, we successfully decreased the expression of IRF9 (Figure 4d), which, in turn, shifted the cells to more mesenchymal phenotypes, presumably by disrupting the ISGF3 complex (Figure 4e). IRF9 KD also significantly decreased the pro-amoeboid effect of IFNβ (Figure S2e). Collectively, our results demonstrate that ISGF3 activity contributes to the maintenance of the amoeboid phenotype and its suppression results in the loss of amoeboid traits and cell elongation.

## 3. Discussion

Here, we have identified a novel mechanism of invasion plasticity regulation governed by IFN signalling mediated by Stat1/Stat2/IRF9 (ISGF3). We show that HT1080 cells induced to undergo MAT upregulate type I IFN signalling (Figure 1). Concordantly, the upregulation of ISGF3 signalling by IFNβ treatment induces MAT in melanoma cells (Figure 2d,e). Notably, the downregulation of ISGF3 signalling by various approaches suppresses the round amoeboid phenotype and leads to cell elongation (Figure 2a–c and Figure 4). Thus, ISGF3 signalling might act as a rheostat favouring either the amoeboid phenotype or the mesenchymal phenotype depending on the activation level, providing cells with an invasion plasticity regulatory mechanism responsive to extra- and intracellular signalling cues.

IFNβ has been demonstrated to exert anti-cancer activities mainly through the attenuation of cell proliferation [35] and the facilitation of anti-tumour immune reaction [26] and has, therefore, been utilized in the treatment of melanoma as a form of cytokine therapy [36]. However, it was also identified as a pro-tumorigenic factor in fibromatosis [37]. Moreover, Stat1, the key effector of the IFNβ signal, was found to play a tumour promoter role in some cases of carcinoma, lymphoma and leukaemia [38]. Proteomic analysis of human triple negative breast tumours revealed that Stat1-positive tumours were more aggressive with increased invasion and lymph node metastasis [39]. Recently, a study analysing three public datasets to identify an epithelial–mesenchymal–amoeboid transition gene signature as a metastasis risk predictor in breast cancer was published [40]. Interestingly, the MAT-associated gene signature derived from this analysis is enriched with type I interferon-response genes (adj. *p*-value 4.3 × 10^−7^) and shows a similar gene enrichment profile as HT1080 cells after MAT (for further information see File S1).

The tumour mass is exposed to various endogenous stress signals, such as hypoxia, nutrient deprivation but also exogenous stress arising in response to chemotherapy and radiation, which all result in upregulated stress signalling, leading to the secretion of various soluble factors, including IFNβ [41]. Moreover, it was demonstrated that direct contact between cancer cells and cancer-associated fibroblasts (CAFs) can lead to the transfer of double stranded DNA from cancer cells to fibroblasts. This interaction triggers the cGAS-STING mediated production of IFNβ in CAFs [42]. This can result in prolonged activation of Stat1/Stat2/IRF9 and, as we suggest, promote amoeboid invasion. In line with this hypothesis, it was recently described that, under hypoxia, epithelial cells undergo a switch from collective to amoeboid invasion [43]. Amoeboid migration is the primordial way of cell migration in metazoans, in an adult organism normally used only by cells of the immune system, but inducible in many (if not all) cancer cells by specific conditions in the tumour microenvironment. Such conditions seem to be de-adhesion from ECM in necrotic areas and dense cell masses, hypoxia, and signalling induced by inflammatory and other ligands released by both the tumour stroma cells and cancer cells themselves [3,43].

There is also increasing evidence that the upregulation of interferon signalling intrinsically present in TME or in response to cell stress promotes the resistance of cancer cells to therapy. IFNβ activated ISGF3 is responsible for constitutive resistance to DNA damage [44]. The upregulation of IRF9 was observed in cells when cultivated in 3D spheroids compared to 2D cultures [45], which was shown to promote resistance to chemotherapeutic drugs [46]. Moreover, IRF9 overexpression leads to resistance to microtubule-targeting drugs [47], and the overexpression of Stat1 is associated with resistance to radiation [48]. IFNβ also promotes the immune escape of glioma cells [49] by increased expression of PDL-1, which we also detected in our samples (Figure 3b).

The precise molecular mechanism underlying the MAT induced by IFNβ is still to be discovered; nevertheless, some clues and parallels can be found in published works. The activation of microglia results in a change in their morphology into an amoeboid shape and an enhanced migratory capacity [50]. This could be among other stimuli also achieved by IFNβ produced by microglia or added exogenously [51]. IFNβ might promote invasion plasticity by upregulating the expression of pro-invasive cytokines, such as IL6 or invasion-associated molecules, such as IFITM1 [52,53,54] or MX1 [55]. Accordingly, we show that treatment with IFNβ promotes the invasion of WM3629 cells from spheroids and increases the number of individually invading cells, most of which utilize the round, amoeboid invasion mode (Figure 3c).

There are several crosstalk mechanisms connecting JAK/STAT signalling to key cytoskeleton dynamics regulators. The ISG15 gene, regulated by Stat1, is one of the crucial effectors of interferon signalling. The ISG15 protein, a small ubiquitin-like covalent modifier, gets attached to numerous target proteins in a process called “isgylation” to affect their activities. ISG15 can disrupt cytoskeletal architecture and promote motility in human breast cancer cells [56,57]. Cytoskeleton remodelling and associated signalling was found to be regulated by the isgylation of IQGAP1 [58,59], non-muscle myosin IIA [60] and filamin B [61]. The activation of Stat1 by focal adhesion kinase FAK is involved in integrin-mediated cell migration and adhesion [62,63]. Cancer cell invasion plasticity is known to be controlled by shifting the balance in the mutually antagonistic regulation of Rac1 and RhoA. Interestingly, the loss of Rac1 in murine keratinocytes decreased actin polymerization and caused the Arp2/3-dependent upregulation of STAT1 and increased interferon sensitivity [64]. Altogether these results show that interferon signalling and Stat1 expression and activity are integrated in a complex regulatory network, also encompassing the actin cytoskeleton.

Here we show that exposure of human melanoma cells to IFNβ may lead to acquisition of the amoeboid phenotype and greatly increase the number of individually migrating cells from spheroids in 3D collagen in vitro. We are aware that these conditions cannot reflect the complexity of TME in vivo, nor can the use of cancer cell lines remove the heterogeneity of cell populations within the tumour microenvironment and crosstalk between malignant and healthy cells. However, various studies have shown that in vitro cultured melanoma cells are a relevant models for primary melanomas [65,66]. Therefore, it may be anticipated that the demonstrated effects of IFNβ on cancer cells kept in 3D culture are paralleled in primary tumours where IFNβ is known to come from several sources. Notably, human melanoma cells were shown to produce IFNβ and are capable of suppressing their own proliferation via the secretion of endogenous IFNβ [36]. It can be speculated that this mechanism could be potentially involved in the switching of human melanoma cells between proliferative and invasive states [67]. Since invasiveness is the most dangerous characteristic of melanoma cells [1], altogether these findings may have important implications for melanoma therapy with IFNβ.

## 4. Materials and Methods

### 4.1. Data Analysis

Transcriptomic data used for the analysis are available from ArrayExpress database at EMBL-EBI under accession number E-MTAB-6823. Only genes significantly affected in both MAT datasets (with criteria FC ≥ 1.5, adjusted *p*-value ≤ 0.25) were selected for the subsequent analysis of gene enrichment using the online tool ShinyGO v0.61 [30].

### 4.2. Cell Lines, Constructs, and Transfection

All cell lines used were of human origin and were routinely cultivated in DMEM medium supplemented by 10% FBS and 10 µg/µL ciprofloxacin (Sigma, Piscataway, NJ, USA) in a humified atmosphere with 5% CO_2_ at 37 °C. Transfections were performed using polyethylenimine (Polysciences, Inc., Warrington, PA, USA) or GenMute transfection reagent (SignaGen Laboratories, Frederick, MD, USA) in the case of DNA and siRNA, respectively, according to manufacturer’s protocol. Stable cell lines were prepared by lentiviral transduction using the second-generation packaging system (pLVX constructs, Tet-On Advanced Gene expression system, Clontech, Mountain View, CA, USA) and enriched by cell sorting, based on EGFP expression. In the case of IFN-treated cells used for morphology studies, RNA and protein lysates, cells were pre-treated with 10 ng/mL recombinant human IFNs (Peprotech, Cranbury, NJ, USA) for 48 hours and IFNs were also present during subsequent 3D cultures (overall exposure time 96 h). Ruxolitinib (Sigma) was used at a concentration of 10 µM, dasatinib (LC Laboratories, Woburn, MA, USA) at 1 µM and doxycycline (Sigma, Piscataway, NJ, USA) at 250 ng/mL. The siRNA sequences targeting IRF9 mRNA were: siIRF9_1: 5′-GCAGAGACUUGGUCAGGUC-3′ and siIRF9_2 5′-CACAGAAUCUUAUCACAGU-3′.

### 4.3. Three-Dimensional Cell Culture and Morphology Analysis

For 3D collagen cultures, cells were mixed with buffer solution and rat tail collagen on ice and plated in wells. After 15 min incubation at 37 °C, the gelled samples were overlaid with cultivation medium containing 1% FBS. The resulting composition of the collagen matrix was 1 mg/mL collagen, 1×RPMI medium, 15 mM HEPES, 1% foetal bovine serum and 50 µg/mL gentamicin. For treated samples, compounds were added to overlaying medium. Cells were cultivated in 3D collagen for 48 hours before further analysis for all applications, except for siRNA treatments that were analysed after 24 h. All images of cells were acquired by Nikon ECLIPSE TE2000-S microscope using Hoffman modulation contrast (10×/0.25 objectives). The morphology of cells was analysed using FiJi software. Cells were considered “elongated” (E) when their length/width ratio was greater than 2, otherwise they were considered “round” (R). A minimum of 100 cells per condition and replicate was analysed and the presented data are summarized from at least 3 independent biological replicates.

### 4.4. Immunoblotting

Protein lysates were prepared from 2D cell cultures, or 3D collagen cultures, where indicated. In the case of 2D samples, cells were harvested and transferred to 1× lysis buffer (1% SDS, 10% glycerol, 60 mM Tris, pH 6.8). For 3D protein lysates, cells were seeded at a density of 1 million cells per 500 µL of 3D collagen gel and cultivated for 48 hours. Gels from two wells per sample were transferred to tubes containing 2× lysis buffer and homogenized using Tissue Tearor (BioSpec Products, Bartlesville, OK, USA). The lysates were processed as described previously [68]. Briefly, lysates were centrifuged, supernatant transferred to new tubes and protein concentration was determined using the DCTM Protein Assay (Bio-Rad Laboratories, Hercules, California, CA, USA) and adjusted to the same protein concentration with 1× SDS lysis buffer. Before SDS-PAGE, DTT (final concentration 50 mM) and bromophenol blue (final concentration 30 µM) were added, and the samples were incubated at 95 °C for 10 min. Samples were run on 10% SDS-polyacrylamide gels and transferred onto nitrocellulose membrane. To prevent non-specific binding, membranes were incubated in TBST with 5% BSA or skim milk and incubated with primary antibodies at 4 °C overnight. The following primary antibodies were used: P-Stat1 (Thermo Fisher Scientific, Waltham, MA, USA; #MA5-15071), P-Stat2 (CST, Danvers, MA, USA; #88410), P-Stat3 (CST; #9145), Stat1 (Thermo Fisher Scientific; #MA5-15129), Stat2 (CST; #72604), Stat3 (CST; #12640), IRF9 (CST; #76684), Jak1 (CST; #3344), MX1 (CST; #37849) and GAPDH (Thermo Fisher Scientific; #MA5-15738). The Western blot images shown are representative of 3 independent biological replicates.

### 4.5. Reverse Transcription–Quantitative Polymerase Chain Reaction (RT-qPCR)

For RNA lysates, cells were seeded at a density of 1 million per 500 µL 3D collagen gel and cultivated for 48 hours. In the case of IFN-treated samples, IFNs were added to overlaying medium (1% FBS). For RNA isolation, two collagen gels per sample were added to tubes containing 600 µL RNA extraction solution (60% *v/v* water-saturated phenol, 3.25 M guanidine thiocyanate, 400 mM sodium acetate buffer pH 4.0, 0.4% *w/v* N-lauroylsarcosine and 160 mM 2-mercaptoethanol) and 100 µL of 6.1 M sodium chloride. Samples were then homogenized using Tissue Tearor (BioSpec Products). RNA was isolated using the modified Trizol method, as described previously [68] and used further for reverse transcription. All RT-qPCR experiments were performed according to MIQE guidelines [69]. For primer details, see Table S1. The Cq values were set by applying a single threshold value for each target using Bio-Rad CFX Manager 3.1 (Bio-Rad Laboratories, Hercules, CA, USA) and exported and further analysed using qBase+ 3.1 software (Biogazelle, Zwijnaarde, Belgium). The list of primers and reference genes is available in Table S1. The obtained data were statistically analysed in GraphPad Prism 6 (GraphPad Software, Inc., San Diego, CA, USA) using paired two-tailed t-test for HT1080 data and one-way ANOVA for WM3629 data.

### 4.6. Three-Dimensional Invasion Spheroid Assay

To obtain spheroids, cells were grown in a 3D Petri Dish^®^ (Microtissues^®^; #12-81 large spheroids, Sigma, Piscataway, NJ, USA) according to manufacturer’s protocol for 2 days in cultivation medium (in case of IFN-treated samples, IFNβ was added). Next, the spheroids were embedded into the 3D collagen matrix and overlaid with cultivation medium. Images of spheroids were taken immediately after embedding into collagen (before) and after 96 hours (after). The area and circularity of the spheroids before and after invasion was assessed using FiJi software (Rasband, W.S., ImageJ, NIH, Bethesda, MD, USA). The data were statistically analysed in GraphPad Prism 6 using one-way ANOVA. Presented data were summarized from 4 independent biological replicates, and at least 4 spheroids per condition, and replicates were analysed.

### 4.7. Proliferation Assay in 3D Collagen

Untreated and IFN-treated cells were seeded into collagen matrix (40,000 cells/100 µL collagen matrix; 4 technical replicates per sample) in a 96-well and cultivated for 48 h. Next, the overlaying medium was replaced by phenol-red free medium containing AlamarBlue reagent (Invitrogen, Carlsbad, CA, USA) in a 5:1 ratio and cultivated for 4 hours. The medium containing AlamarBlue was then transferred to new wells and fluorescence (excitation 550 nm; emission 590 nm) was measured using the Infinite M200 Pro plate fluorimeter (TECAN, Mannedorf, Switzerland). Collagen without cells served as a blank for the experiment. The results are summarized from 3 independent biological replicates. The data were statistically analysed in GraphPad Prism 6 using one-way ANOVA.

## 5. Conclusions

Collectively, previous studies and our results suggest that, in response to stress, metastatic cells may activate IFN associated signalling and gain a rounded amoeboid phenotype. We show that although IFNβ decreases cancer cell proliferation, it promotes invasion plasticity, which can endow cancer cells with an escape mechanism from local stress inducing conditions. In summary, we point out the role of IFNβ activated Stat1/Stat2/IRF9 signalling in cancer invasion plasticity, aside from its known role as a tumour suppressor.

## Supplementary Materials

The following are available online at https://www.mdpi.com/2072-6694/12/9/2450/s1, Figure S1: Detailed information about Western blot in Figure 1, Figure S2: Additional Characterization of IFN treated cells, Figure S3: Detailed information about Western blot, Figure S4: Detailed information about Western blot in Figure 2, Figure S5: Detailed information about Western blot in Figure 3, Figure S6: Establishment of WM3629 stable cell lines expressing EGFP-tagged doxycycline-inducible SOCS1 and SOCS3, Figure S7: Expression of iSOCS1 can prevent dasatinib-induced MAT in HT1080 cells, Figure S8: Detailed information about Western blot in Figure 4, Table S1: RT-qPCR primers used in the work, Video S1: WM3629 IFNb. File S1: Gene Ontology Enrichment Analysis.
