# Supplementary material for: Sustained Inflammatory Signalling through Stat1/Stat2/IRF9 Is Associated with Amoeboid Phenotype of Melanoma Cells
Source: Cancers (Basel). 2020 Aug 28;12(9):2450. doi: 10.3390/cancers12092450 (PMC7564052; doi:10.3390/cancers12092450)
Supplement: Supplementary file 1 [file cancers-12-02450-s001.zip › cancers-879867 - final - supplementary/cancers-920664 - supplementary.pdf]

Supplementary Materials

Sustained Inflammatory Signalling through Stat1/Stat2/IRF9 Is Associated with the Amoeboid Phenotype of Melanoma Cells

Aneta Gandalovičová, Anna-Marie Šůchová, Vladimír Čermák, Ladislav Merta, Daniel Rösel and Jan Brábek

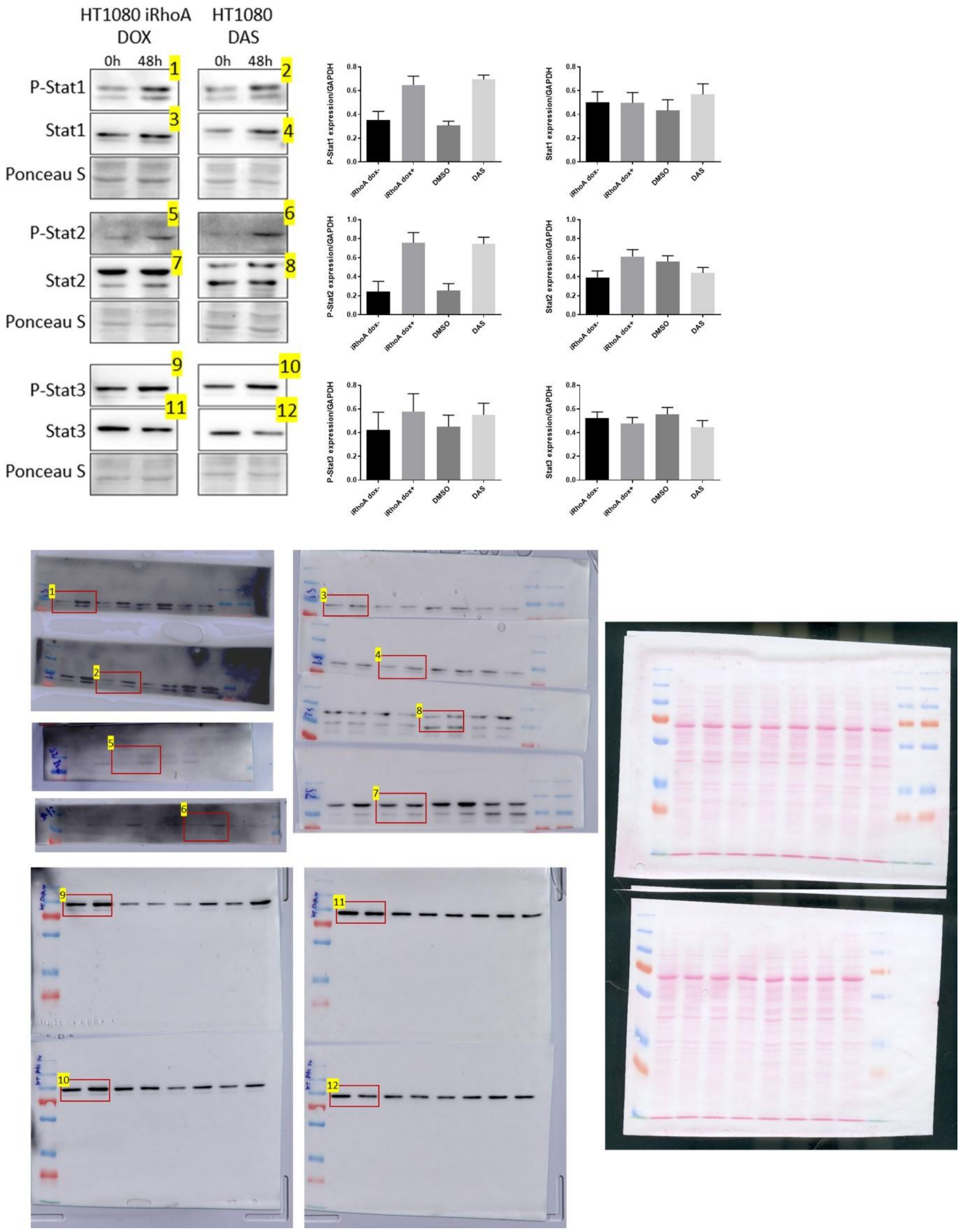

Figure S1. Detailed information about western blot in Figure 1.

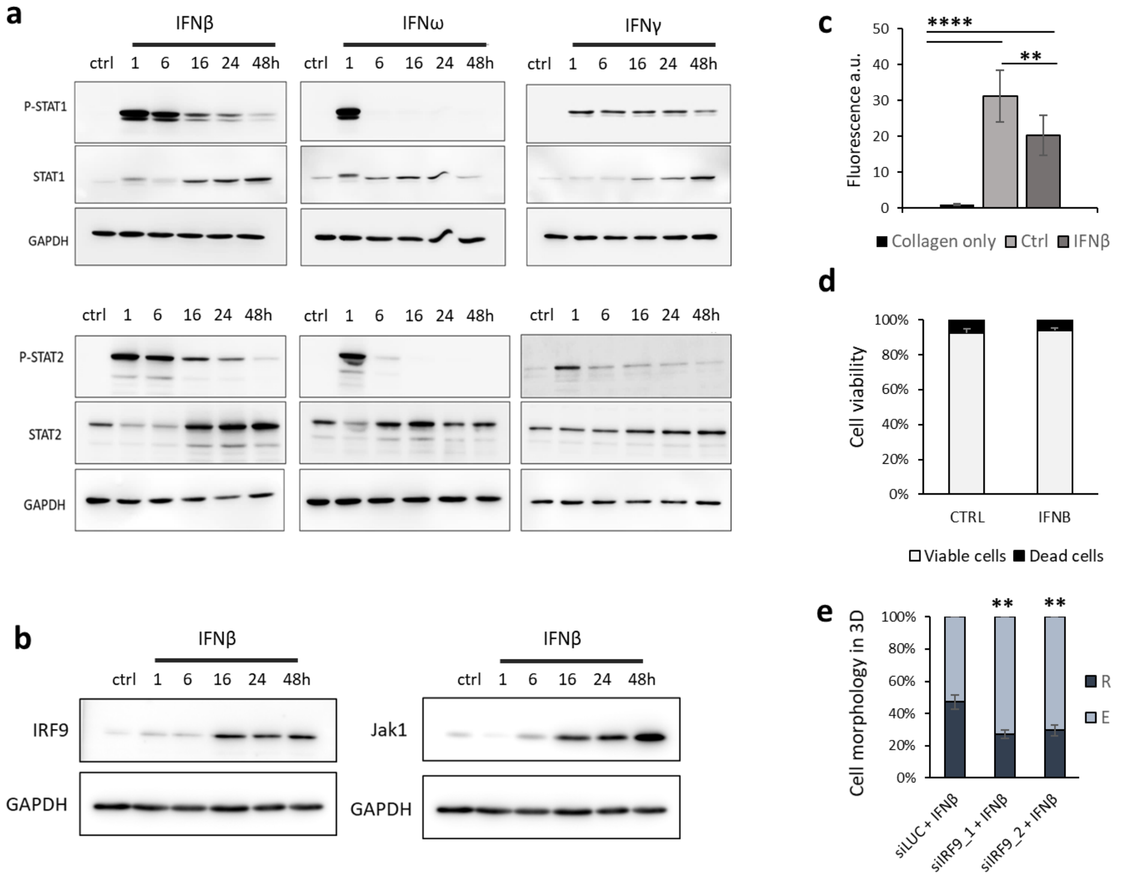

Figure S2. Additional Characterization of IFN treated cells (**a**) Immunoblotting for the activation of Stat1 and Stat2 in time course samples of WM3629 cells treated with IFNs. (**b**) Immunoblotting for IRF9 and Jak1 in time course samples of WM3629 cells treated with IFNβ. (**c**) Proliferation of control and IFNβ treated WM3629 cells in 3D collagen after 48 h measured by modified Alamar Blue assay. (**d**) Cell viability of control and IFNβ treated WM3629 cells after 48 h assessed by trypan blue exclusion assay. (**e**) Quantification of morphology of WM3629 cells with siRNA KD of IRF9 treated with IFNβ in 3D collagen. *p*-values: ***** $p < 0.0001$, ** $p < 0.01$.*

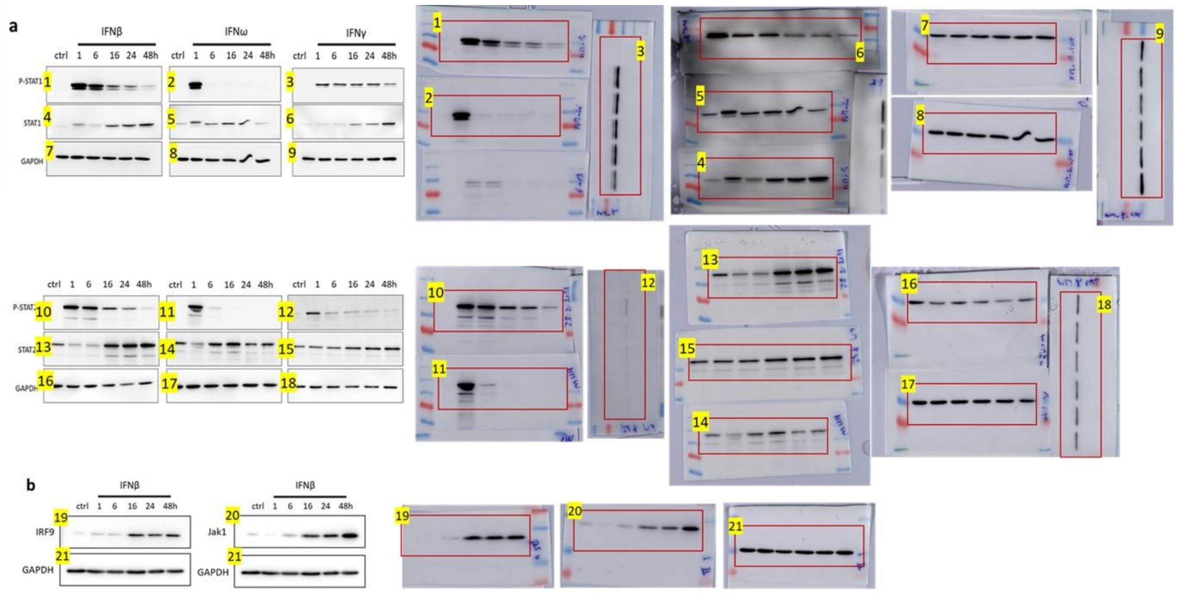

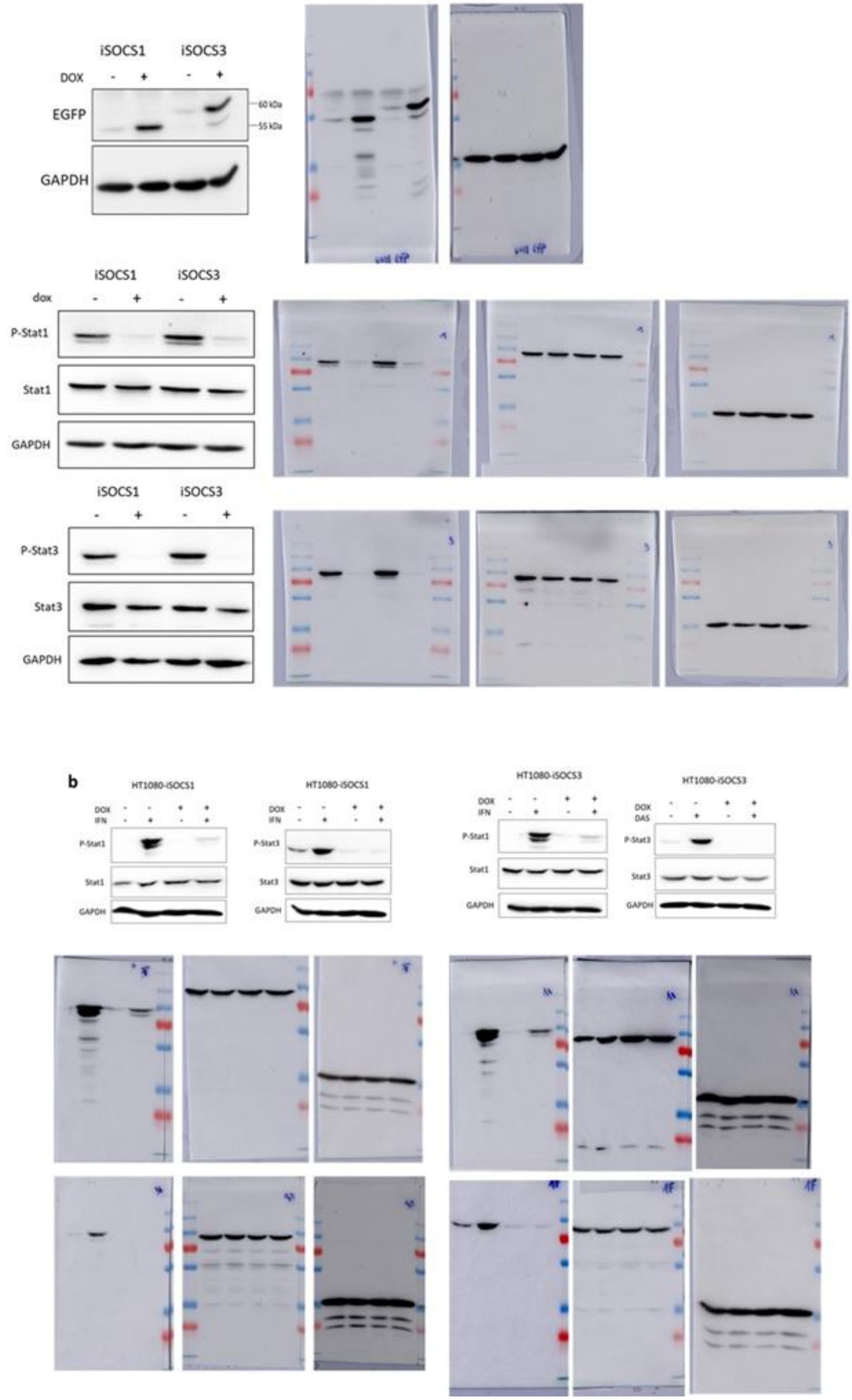

Figure S3. Detailed information about western blot.

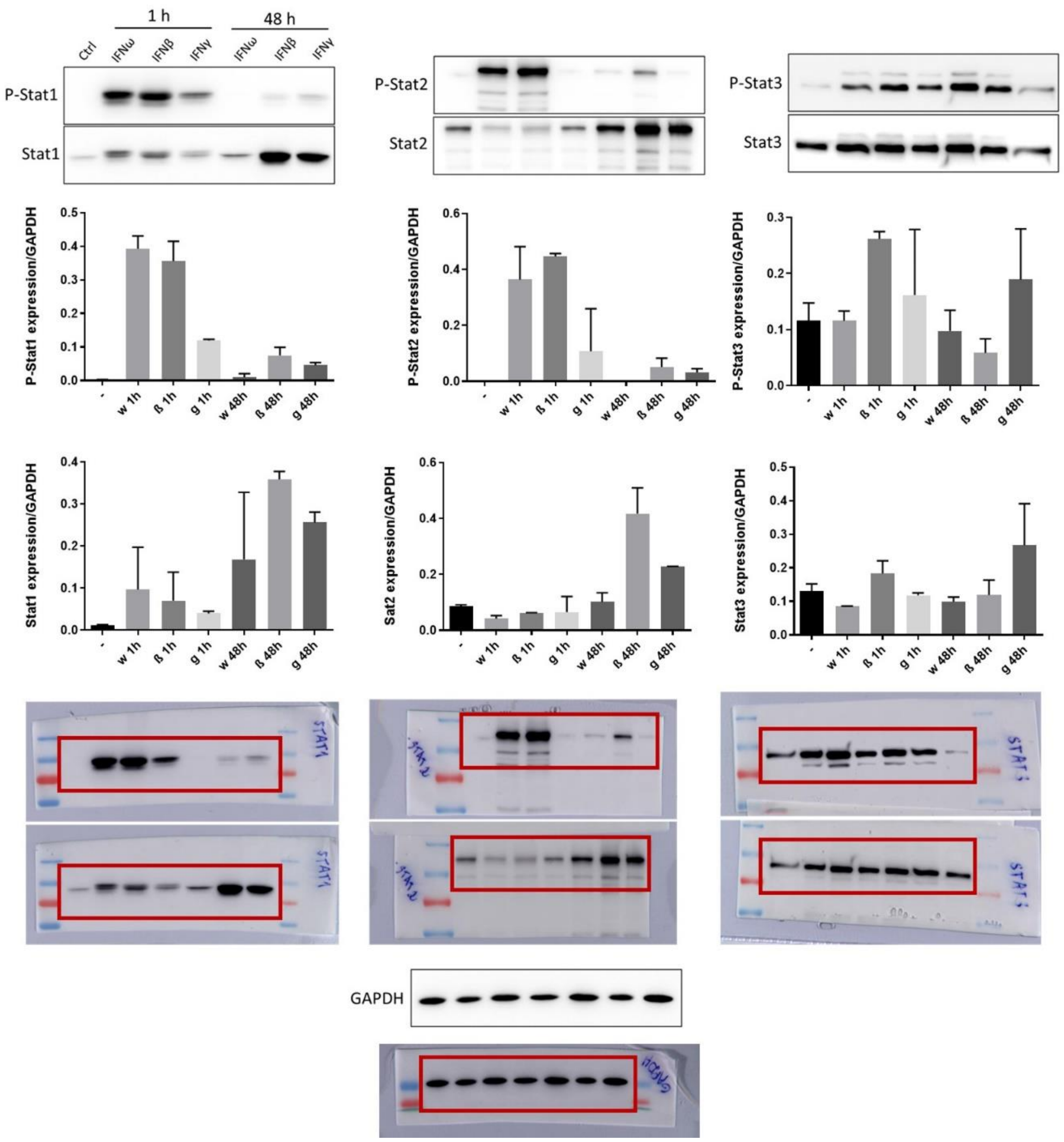

Figure S4. Detailed information about western blot in Figure 2.

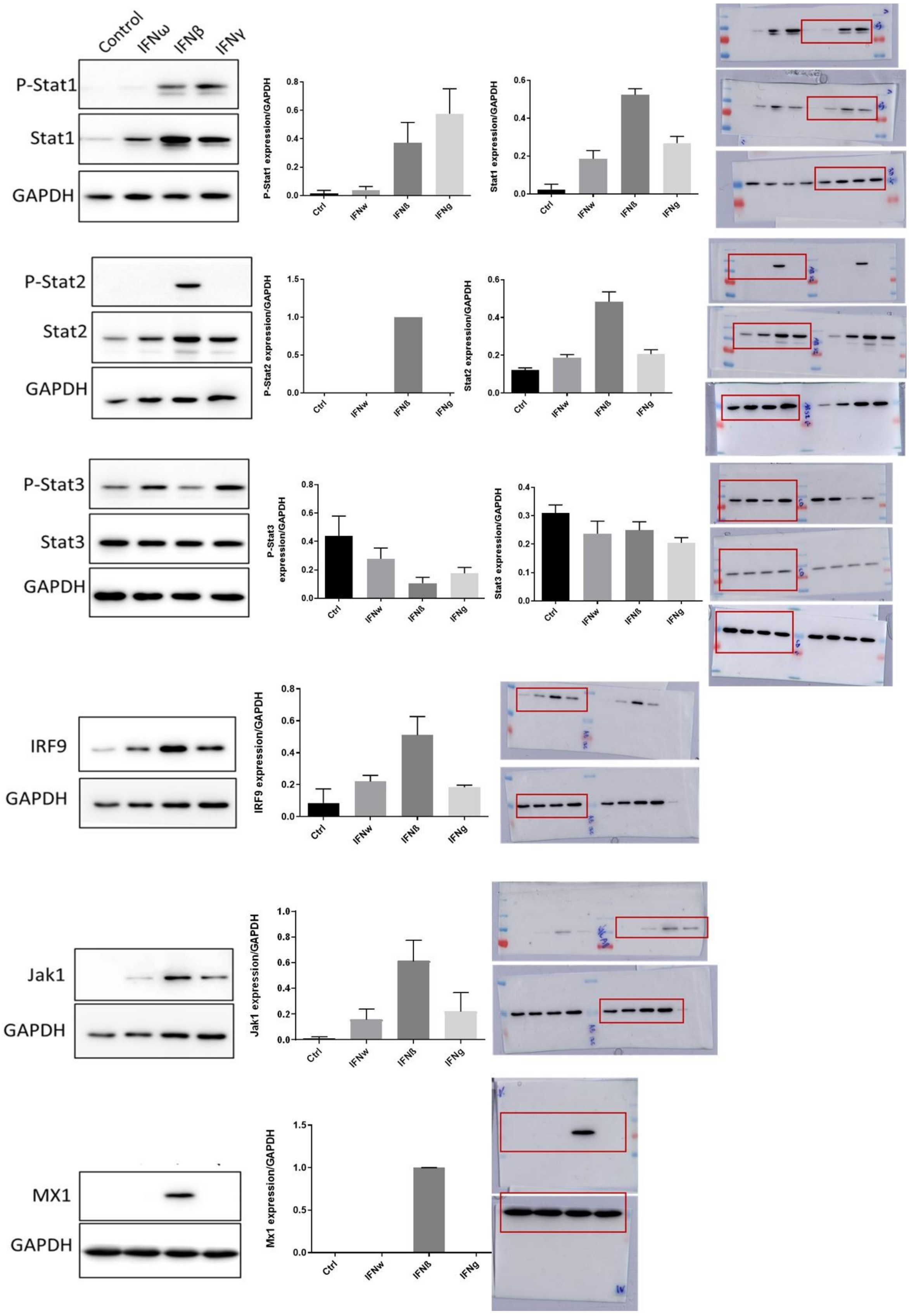

Figure S5. Detailed information about western blot in Figure 3.

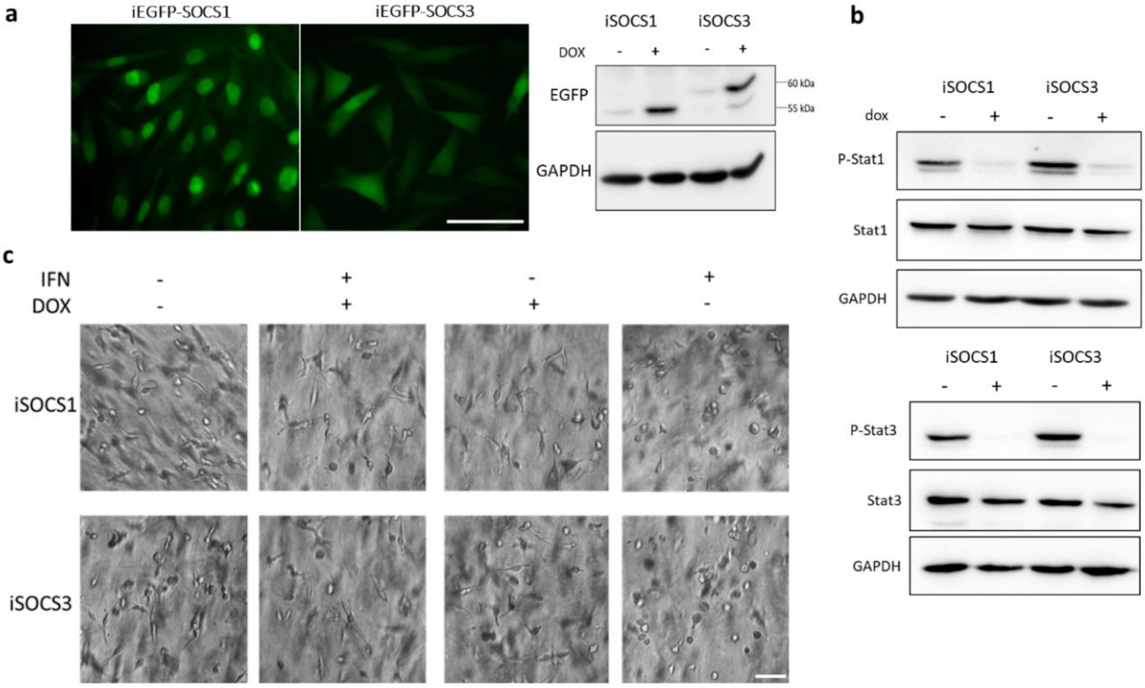

Figure S6. Establishment of WM3629 stable cell lines expressing EGFP-tagged doxycycline-inducible SOCS1 and SOCS3. (**a**) Validation of expression of inducible SOCS1 and SOCS3 (iSOCS1, iSOCS3) visualized by fluorescence microscopy of EGFP signal after addition of doxycycline (DOX) (left) and by immunoblotting for EGFP. (**b**) Immunoblotting for Stat transcription factors Stat1 and Stat3 activation after induced expression of iSOCS1 and iSOCS3 in WM3629 cells. (**c**) Representative image of WM3629 stable cell lines expressing iSOCS1 and iSOCS3 treated with IFNβ without or with induced expression of iSOCS1 and iSOCS3 in 3D collagen. Scale bar 100 µm.

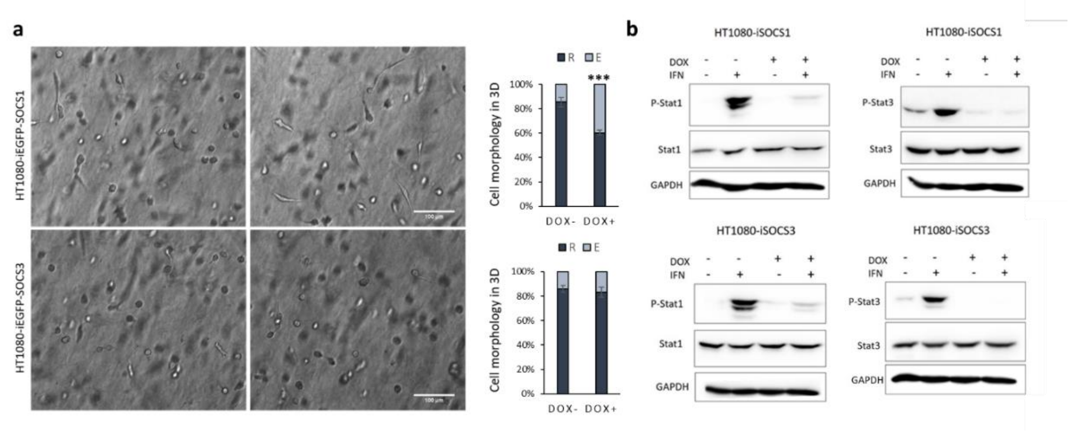

Figure S7. Expression of iSOCS1 can prevent dasatinib induced MAT in HT1080 cells**.** (**a**) Representative image of HT1080 stable cell lines expressing EGFP-tagged doxycycline-inducible SOCS1 and SOCS3 (iSOCS1, iSOCS3) treated with dasatinib (DAS) to induce MAT without or with induced expression of iSOCS1 and iSOCS3 in 3D collagen and quantification of cell morphology. (**b**) Immunoblotting for Stat transcription factors Stat1 and Stat3 activation after IFN treatment and induced expression of iSOCS1 and iSOCS3 in HT1080 cells. Scale bar 100 µm. R = round, E = elongated. *p*-values*:* *** $p < 0.001$.

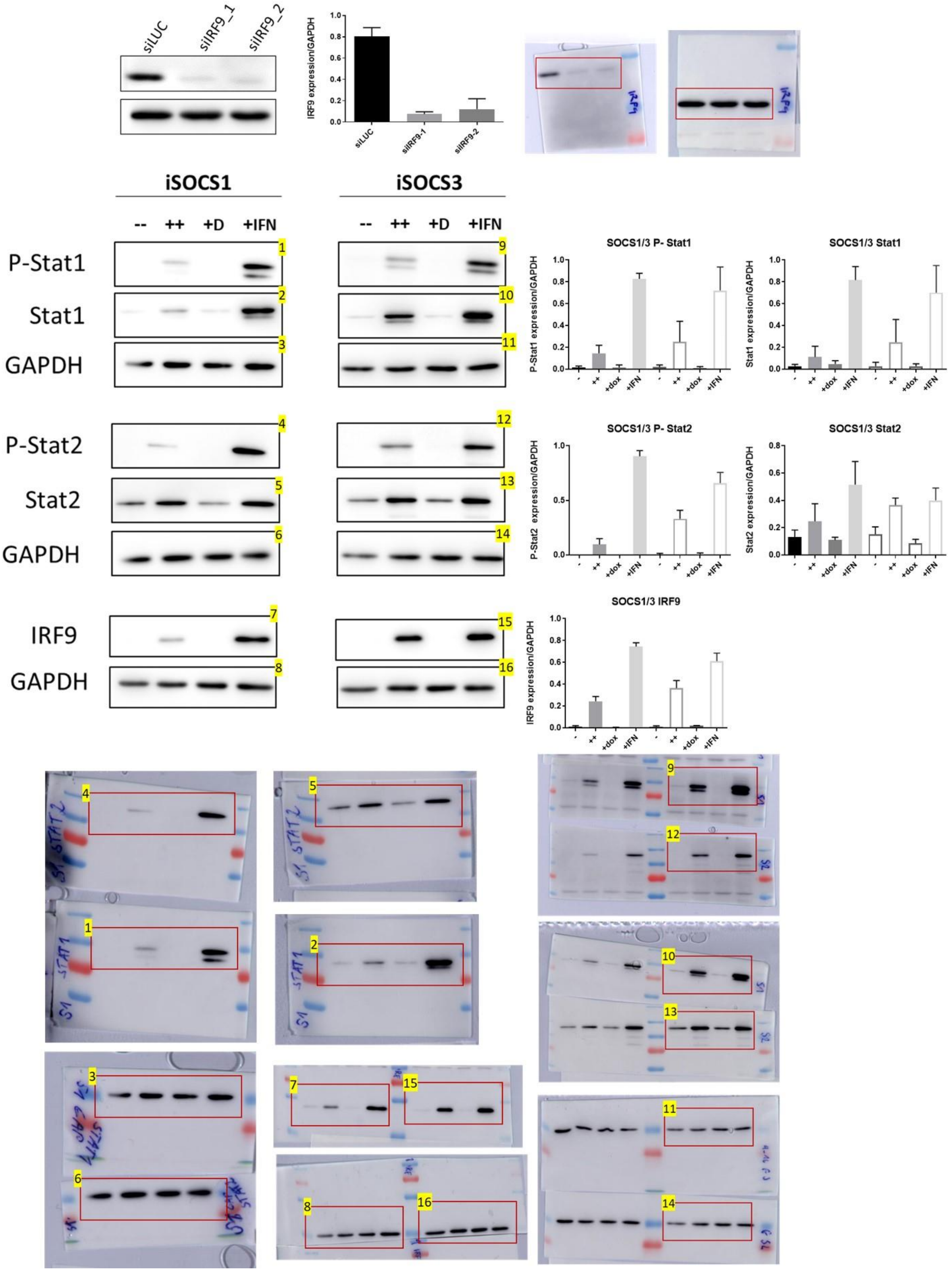

Figure S8. Detailed information about western blot in Figure 4.

Table S1. RT-qPCR primers used in the work.

Target Gene	Forward Primer	Reverse Primer
STAT1	CTGAATTTCGGCACCTGCAAT	GCTCTTCAGTAACGATGAGAGGA
STAT2	GGGACTTTGGTTACCTGACTCT	CAGTTCCTCTGTCACACCTAGT
STAT3	AGTGTGCATTGACAAAGACTCTG	CGTTGTTGGATTCTTCCATGTTC
IRF9	AGCTCTTCAGAACCGCCTAC	CAGTGTTACCTGGAACTTCGGT
MX1	CACAGAACCGCCAAGTCCAA	ACCTGGTCCTGGCAGTAGAC
ISG15	GAGAGGCAGCGAACTCATCT	CTTCAGCTCTGACACCGACA
IL6	GTTCCTGCAGAAAAAGGCAAAGA	GTCAGCAGGCTGGCATTTGT
IL8	CACACTGCGCCAACACAGAAA	CTCAGCCCTCTTCAAAAACTTCTC
IL24	CCTGTGGACTTTAGCCAGACC	GCAGGGTAAAACCCAGGCAA
IFNB1	CTAGCACTGGCTGGAATGAGAC	CCAGGACTGTCTTCAGATGGTTT
IFITM1	TCAACACCCTCTTCTTGAACTGG	CTTCCTGTCCCTAGACTTCACG
PDL1	CTGGCATTTGCTGAACGCAT	TTAGTGCAGCCAGGTCTAATTGT
Reference gene (HT1080)		
EIF4H	CCTTCTGGCTACGGGAACCAT	CTTCGACACCTACGACGATCG
GAPDH	GCATGGACTGTGGTCATGAG	CTGCACCACCAACTGCTTAG
Reference gene (WM3629)		
HNRNLP	GCGCTCACTTTTGCCTGAGAA	CTGGAGGTGACCGAGGAGAA
PPIA	GCCGAGGAAAACCGTGTACTA	CTGCAAACAGCTCAAAGGAGAC

© 2020 by the authors. Licensee MDPI, Basel, Switzerland. This article is an open access article distributed under the terms and conditions of the Creative Commons Attribution (CC BY) license (http://creativecommons.org/licenses/by/4.0/).
